# In Vitro Gametogenesis in Oncofertility: A Review of Its Potential Use and Present-Day Challenges in Moving toward Fertility Preservation and Restoration

**DOI:** 10.3390/jcm12093305

**Published:** 2023-05-06

**Authors:** Victoria G. Wesevich, Christopher Arkfeld, David B. Seifer

**Affiliations:** 1 Division of Reproductive Endocrinology and Infertility, Department of Obstetrics, Gynecology & Reproductive Sciences, Yale School of Medicine, New Haven, CT 06510, USA; 2 Department of Obstetrics, Gynecology & Reproductive Sciences, Yale New Haven Hospital, New Haven, CT 06510, USA

**Keywords:** oncofertility, fertility preservation, in vitro gametogenesis, induced pluripotent stem cells, assisted reproductive technology, medical ethics, reproductive rights

## Abstract

Current fertility preservation options are limited for cancer survivor patients who wish to have their own biological children. Human in vitro gametogenesis (IVG) has the hypothetical ability to offer a unique solution to individuals receiving treatment for cancer which subsequently shortens their reproductive lifespan. Through a simple skin punch biopsy, a patient’s fertility could be restored via reprogramming of dermal fibroblast cells to induced pluripotent stem cells, then from primordial germ cell-like cells into viable oocytes and spermatocytes which could be used for embryogenesis. Induced pluripotent stem cells could also be used to form in vitro environments, similar to the ovary or testes, necessary for the maturation of oogonia. This would allow for the entire creation of embryos outside the body, ex vivo. While this area in stem cell biology research offers the potential to revolutionize reproduction as we know it, there are many critical barriers, both scientific and ethical, that need to be overcome to one day see this technology utilized clinically.

## 1. Scope of the Issue

Fertility preservation remains one of the most important and dynamic areas of focus for reproductive endocrinology and fertility specialists. There are approximately 1.9 million people diagnosed with cancer each year in the United States (US), the most frequent being breast cancer with nearly 290,000 people diagnosed per year [1]. In the US, there are about 70,000 adolescent and young adults, aged 15–39 years old, diagnosed with cancer each year [2]. Modern improvements in cancer screening and therapies have decreased overall mortality of cancer increasing cancer survivorship, and thus, placing an emphasis upon quality of life for cancer survivors. The current estimate is that there will be 26 million cancer survivors by 2040 [3]. Present-day chemotherapy, radiation, and/or surgical treatment of cancer often acutely shortens the longevity of reproductive potential of many of these young people. Thus, preservation of fertility is a meaningful quality of life and survivorship issue amongst cancer survivors [4].

Unfortunately, current fertility preservation options are limited for cancer survivor patients of reproductive age. Current female fertility preservation options include suppression of the hypothalamic–pituitary–ovarian axis with gonadotropic-releasing hormone (GnRH) agonists, as well as oocyte, embryo (requiring sperm donor), and ovarian tissue cryopreservation. Certain cancer diagnoses require urgent initiation of treatment, in which cryopreservation of oocytes or embryos risks delaying initiation of chemotherapy due to the time required to complete controlled ovarian hyperstimulation [5,6]. In addition, some patients are not appropriate surgical candidates at the time of cancer diagnosis, such as pancytopenia in the setting of hematologic cancers. In these instances, even the quicker process of ovarian tissue cryopreservation surgery is also not feasible. Similar challenges are faced by male patients. Post-pubertal male patients can undergo sperm banking. Oncologic testicular sperm extraction (TESE) is also available in patients who are unable to produce a semen sample or have aspermia, resulting in about a 50% successful sperm retrieval [7]. This still leaves a significant number of patients who are unable to undergo fertility preservation prior to initiation of gonadotoxic cancer treatment. Additionally, it has been consistently found that many patients are under-counseled and ultimately do not undergo fertility preservation [8,9]. The financial restraints of out-of-pocket expense of current fertility preservation options creates additional barriers for patients, thus limiting the number of patients that are able to undergo fertility preservation in a critically urgent manner [10].

An exciting area of current research which may one day help to overcome these obstacles is human in vitro gametogenesis (IVG). IVG is the process of making gametes outside the body. IVG uses either 46XX or 46XY somatic cells to make an oocyte or sperm cell from those somatic cells in the lab and fertilizing them with gametes of the opposite biologic sex in order to make an embryo. The ability to generate gametes without the use of natural gametes presents a unique opportunity within reproductive medicine with particular applications for the field of oncofertility as well as for possible future opportunities of non-gamete dependent reproduction using induced pluripotent stem cells (iPSC).

While IVG holds great potential, quality measures have yet to be established to safeguard from its inappropriate advancement. Therefore, an important area of future investigation is to ensure the biologic integrity of oocytes in each stage of maturation in IVG. This will require careful validation studies for the integrity of each step of the process. Given the restrictive environment of human embryology research, initial studies will occur in mouse models, testing the ability of oocyte in vitro maturation (IVM) to develop from blastocysts into healthy pups. Despite decades of research, IVM has been a particularly challenging hurdle for which there remains no widely adopted clinical technique. Continued IVG research will enhance our current understanding of assisted reproductive technology (ART). This includes potentially improving outcomes for patients with infertility who would not require IVG to procreate. For example, if a patient with cancer wishes to preserve her fertility prior to chemotherapy, radiation and/or surgery, they may elect to proceed with tissue cryopreservation followed by surgical autologous transplantation which would be required for oocyte maturation. IVG research could strengthen our understanding of IVM to the point where primordial follicles from cryopreserved ovarian tissue could be used for the in vitro creation of embryos without the risk of reimplantation of potential cancer cells back into the patient.

## 2. Promise of Human In Vitro Gametogenesis (IVG)

Human IVG has the potential to offer a unique solution to individuals receiving treatment for cancer which subsequently shortens their reproductive lifespan. Once available clinically, IVG could allow patients to first focus on their cancer treatments without additional medications, procedures or surgeries between time of diagnosis and initiation of treatment. These patients would have a skin punch biopsy performed and preserved for subsequent IVG to restore their reproductive potential. Therefore, this method could address several of the shortcomings of current fertility preservation methods. One patient population with unique limitations in fertility preservation is the pre-pubertal cancer patient. In males, spermatozoa are not formed until puberty, making the collection of semen used for sperm cryopreservation not possible. Pre-pubertal testicular tissue preservation remains experimental, as there have not been live births seen as a result of this technique. In pre-pubertal women, oocytes are not responsive to the medications used for oocyte maturation used in in vitro fertilization (IVF). This excludes them from the commonly utilized option of controlled ovarian hyperstimulation followed by oocyte cryopreservation for fertility preservation in post-pubertal women. IVG could offer a unique solution to pre-pubertal males and a less invasive alternative to ovarian tissue cryopreservation in pre-pubertal females. IVG could theoretically be performed after completion of a patient’s cancer treatment, thereby allowing patients additional time to plan for the acquisition of resources required for treatment; thus, making IVG a more widely available and timely solution when they are less likely to be distracted by a new cancer diagnosis and its current possibility of a childless future.

In this review, we summarize the current state of IVG, the various optional methodologies that could be utilized as well as future necessary research to achieve its promising applications in preservation and possible restoration of fertility in cancer survivors. We also explore some of the ethical concerns associated with this transformative approach.

## 3. Human IVG

Mammalian gametogenesis is the process of germ cells differentiating into dimorphic gametes, oocytes, and spermatozoa. The haploid female oocyte fuses with the haploid male spermatozoon to form a diploid zygote which subsequently begins embryogenesis [11]. In order to facilitate embryogenesis, germ cells must maintain totipotency at the time of fertilization to appropriately develop as embryos. IVG aims to replicate normal germ cell development from lab-differentiated cells. Stem cells retain the ability to differentiate into any cell of an organism. As stem cells further differentiate, they specialize and lose their developmental potency. Ultimately, stem cell differentiation concludes as a unipotent cell of the organism.

One form of IVG utilizes embryonic stem cells (ESCs) collected from the inner cell mass of the blastocyst after fusion of the female oocyte and the male spermatozoon. These cells are pluripotent and can develop into cells of all germ layers but not extraembryonic structures (i.e., placenta). These cells must be collected before implantation. The process of culturing ESCs is conducted by collecting cells from the inner cell mass of the blastocyst on approximately day 4 of development after fertilization. Isolation and collection of these ESCs typically takes place in an embryology lab specialized in in vitro fertilization. Alternatively, induced pluripotent stem cells (iPSCs) are obtained after implantation. iPSCs are created through the introduction of genes into somatic cells, inducing de-differentiation (known as reprogramming), causing them to become pluripotent (Table 1) [12].

Adult stem cells (ASCs) are found in differentiated tissue that retains the ability to renew themselves and generate new cells in response to damage or dying cells. These are synonymous with somatic stem cells (SSCs). The major distinction is that both ASCs and SSCs are non-reproductive cells (i.e., not oocytes or spermatozoa). One well-known multipotent stem cell line consist of hematopoietic stem cells (HSC), which are used in bone marrow transplant patients to replete and replace malignant blood cell lines [13]. The HSCs have the ability to regenerate blood cell lines and offer the framework to understand different cell lines’ regenerative ability. The process of reprograming ASCs into PSCs requires transferring the ASC nucleus into the cytoplasm of an oocyte. This somatic cell nuclear transfer was the process used to clone Dolly the Sheep. This same process has now been used in non-human primate studies as well [14].

Reprogramming differentiated somatic cells into induced pluripotent stem cells (iPSCs) relies on cell reprogramming. In a mouse model, reprogramming focuses on downregulation of p53 and increase of oncogenes Myc and Klf4 (Kruppel-like factor 4). However, in that mouse model, these changes may increase the risk of mutations and could possibly lead to cancer or congenital birth defects in the developed organism [15]. To reprogram somatic cells, the epigenome must also be reconstructed using transcription factors and genomic methylation [16]. To achieve clinical utility, stem cells must differentiate into the desired cell types free of mutations. This is controlled via cellular culture manipulation. The current differentiation protocols mirror gastrulation, differentiating into ectodermal, mesodermal, and endodermal progenitors.

In 2011, the first successful in vitro development of mouse haploid sperm and mouse haploid oocytes from iPSCs was completed [17]. This scientific advancement, in conjunction with developing ovarian tissue and testicular tissue cryopreservation techniques, increased the potential clinical applications of iPSCs therapy. Specifically, patients receiving gonadotoxic chemotherapy could potentially benefit from transplantation.

Functional oocytes have been generated from pluripotent stem cell-derived primordial germ cell-like cells (PGCLCs) when cultured with embryonic ovarian somatic cells in a mouse model [18]. The signaling process that takes place causing cellular differentiation includes physical stimuli from nearby cells, chemical signaling between cells, and internal signals generated by cellular DNA [12]. The use of iPSCs from somatic cells addresses many of the ethical conflicts related to the use of embryonic stem cells. Since these cells are autologous, the recipient does not require immunosuppressive management to prevent stem cell rejection.

## 4. Current Status of IVG Research

### 4.1. Animal Models

IVG research in animal models may serve to benefit both animals and humans. It has been proposed that IVG may be a solution to prevent endangered species from becoming extinct [19]. For example, similar processes have been applied most recently with the goal of helping prevent the extinction of the white rhinoceros [20]. Stem cell and embryology research using animal models is not bound by tight regulations, allowing us to explore the biologic pathways to ultimately be applied to human research. Given the limitations on this research in humans (reviewed below), animal models will likely be the first to demonstrate the application of IVG to the ‘cancer survivor patient’.

### 4.2. Mouse Model

The first successful IVG from pluripotent stem cells using a mouse model was performed in 2011 [17]. Since then, research has continued to evolve and now focuses on IVG from somatic cells derived from tail-tip fibroblasts. Development of oocytes from somatic cells may be preferred over primordial germ cells since primordial germ cells are more challenging to obtain from both a logistic and ethical perspective. The entire process of IVG takes approximately 4–5 months for in vitro maturation and is dependent on specific culture systems with genes (*Figla, Sohlh1, Lhx8, Nobox, Stat3, Tbpl2, Dynll1* or *Sub1*), which have been shown to drive the primordial to primary oocyte transition and prompt the capacity for oocyte growth [21,22]. One observed difference in mouse models, in contrast to potential human IVG, is that mouse oocytes do not arrest in meiosis and are all able to be fertilized. The transition of primordial to primary follicle has been described as one of the most important areas of research to have advanced the field.

A unique aspect about oocyte development is that oocytes require ovarian stromal, granulosa, and theca cellular support to progress to a stage in which fertilization is possible [21]. Oogonia develop from primordial germ cells in fetal gonads. These oogonia mature into oocytes with the help of ovarian stromal cells which ultimately form cumulus–oocyte complexes at the time of ovulation. These are made of a secondary oocyte arrested at metaphase II following extrusion of the polar body as well as its surrounding cumulus cells. Currently, this entire process has been completed in an in vitro mouse model, where all cells of the cumulus–oocyte complex were reprogrammed from induced pluripotent stem cells (iPSC).

Although in vitro oogenesis has proven to be especially complex, the female germ cell was cultured in vitro utilizing mouse pluripotent stem cells derived from tail-tip fibroblasts [22]. One major accomplishment has been the birth of healthy-appearing pups utilizing fetal ovarian somatic cell-like cells (FOSLCs), via reconstituted Ovarioids (rOvarioids) mimicking follicles, aggregated with PGCLCs derived from mESCs into oocytes able to mature and be fertilized. These mature cumulus–oocyte complexes were fertilized with wild-type sperm from mice. From an initial 996 mature oocytes, 24 two-cell embryos were successfully developed into blastocytes. All live pups grew into adult mice without obvious birth defects or cancer [18].

The first reported functional spermatids were generated in vitro from ESC-derived PGCLCs that were subsequently differentiated into haploid SLC that were successfully used in intracellular sperm injection for fertilization in 2016. Importantly, in this study, they established a protocol for in vitro spermatic meiosis and gametogenesis [23]. In 2021, in vitro spermatogenesis was accomplished from mESC induction to mPGCLC to spermatogonium-like cells (SLC), matured in vitro using reconstituted (r)Testis [24]. The genetic and epigenetic properties of the generated SLC need investigation. Studies of both IVG-created oocytes and sperm have each resulted in the birth of live mouse pups when combined with the wild type of the opposite gamete. The genetic, epigenetic, and as well as behaviors, metabolic profile, and disease susceptibility have not been studied in the offspring.

### 4.3. Non-Human Primate Model

Translating these aforementioned findings into human embryo research has been aided by non-human primate models. Monkey models are more biologically similar to humans but are not under the same legal restrictions as human embryo research [25]. Thus far, PGCLC differentiation has been successfully performed in rhesus macaques from rhesus iPSCs. At this time, following xenotransplantation in the mouse or homologous transplantation into a monkey, these PGCLCs were not able to be ultimately reprogrammed into spermatogonia, but only able to achieve late rhesus PGCs [26]. Similarly, in cynomolgus monkeys, early PGCs have been derived from iPSC from ESCs.

### 4.4. Human Studies

Transitioning from animal to human studies evokes a host of relevant ethical and regulatory concerns. New guidelines regarding human embryo research were published in May of 2021. This included research endeavors of human embryos beyond 14 days to be considered for removal from the ‘prohibited’ category, and an open public discussion on the permissiveness of such activities was encouraged [27,28]. This type of research is still illegal in the US, and the International Society for Stem Cell Research (ISSCR) guidelines have not yet moved this research to a ‘permissive’ category. Thus, such discourse of human use has been initiated to consider changing and modifying the current restrictions on embryo research. To present day, all human embryo research worldwide in IVG has been conducted under this 14-day restriction.

**Figure 1:**
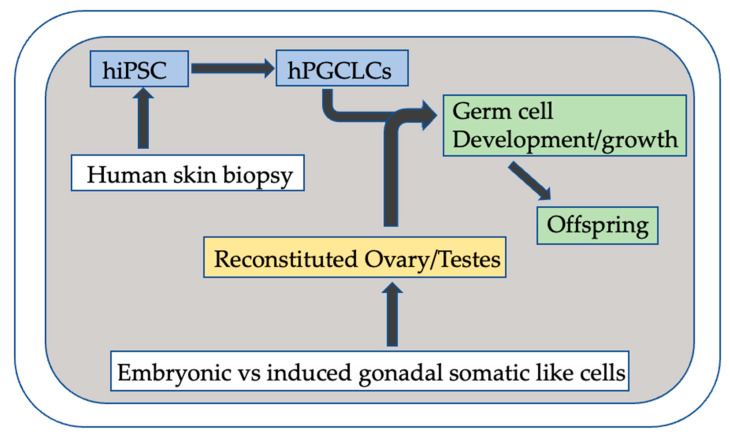
Human In Vitro Gametogenesis-Simplified Schematic. iPSC: human induced pluripotent stem cell, miPSC: mouse induced pluripotent stem cell, hPGCLC: human primordial germ cell like cell.

Over 15 years ago, a pathway for differentiated human somatic cells (fibroblasts) to be reprogrammed into pluripotent cells using specific transcription factors was described [29]. Fast forward to today, and researchers have been able to differentiate hiPSC into both early oogonia and prospermatogonia. This maturation occurs in an air–fluid interface of mouse embryonic somatic cell-derived xenogenic reconstituted ovaries (xrOvaries, similar to rOvarioids) and xrTestis, respectively [18,30,31,32]. Importantly, it is thought that if the artificial ovary and testes culture systems could be derived from human somatic cells, the differentiation of human gametes would be more efficient. Currently, the mouse-derived environment takes 70 days to show some critical genes as evidence of maturation of oogonia. The xrOvaries also take 120 days to demonstrate partial X reactivation and important methylation changes [31]. The specific human somatic cells capable of supporting such differentiation are difficult to acquire. Therefore, the goal is to be able to induce patient-specific ovarian/testicular cells, also using dermal fibroblast cells (Figure 1).

Currently, experiments using human models would be halted at the blastocyst stage, as there are federal laws limiting the study of embryos beyond 14 days [28]. Under current regulations, IVG-derived germ cells would require validation studies relative to naturally occurring germ cells in an effort to demonstrate feasibility, effectiveness, and safety.

Such validation studies are already beginning via human twin studies. Recent published works examined developing hiPGCLCs from hiPSCs from monozygotic (MZ) monoamniotic (MA) twins with discordant primary ovarian insufficiency (POI) [33,34]. These studies identified that both the fertile and POI twins had equivalent potential to form hiPSC. This provides evidence to support that discordant POI found in MZ, MA twins is most likely a timing issue leading to disproportionate allocation of the primordial germ cell progenitor pool, resulting in POI in the under-allocated twin. Additionally, the authors found that all hiPSC sublines had more genomic changes than expected. This raises concern that prior to differentiation into oogonia, the induced cells are non-equivalent to those found in nature at a genetic level. This situation would complicate reaching the ultimate goal of safe embryo development and thus necessitates further study. Germ cells developed from each adult twin’s somatic cells retained similar ability for germ cell production. This affirms the utility of targeting somatic cells as the source for IVG.

Furthermore, there are many additional active areas of IVG research that continue to contribute to our understanding of human embryos. Such examples include studies of human primordial germ cell development including X-chromosome inactivation versus dampening, a phenomenon unique to pre-implantation embryos [35]. This may serve as a genetic marker and means of developmental stage and quality assessment. Another study, not utilizing hiPGCLC (oogonia and spermatogonia), has been able to study blastocyst-like cells—iBlastoids, which have been derived from somatic cells. iBlastoids, while not capable of turning into a fetus, do undergo the early stages of embryogenesis. This will allow for another avenue for investigation of implantation using hiPSC from human dermal fibroblasts [36]. Additionally, there has been the derivation of a human trophoblast cell line, an extra-embryonic tissue (placenta) [37]. This cell line offers novel potential for the study of a particularly challenging and poorly understood area of study of early pregnancy. It will provide a new avenue of study for human trophectoderm and trophoblast development, as well as early cell-fate decisions in embryogenesis.

## 5. Future of IVG: Possible Implications for Oncofertility and Beyond

At present, a major barrier within the human application of IVG is forming functional cells beyond the early stages of oogenesis in order to successfully complete meiosis and folliculogenesis [38]. While studies on early oogonia created from iPSC have developed oocytes that appear normal, these early oocytes are too immature to become fertilized and thus currently cannot become embryos. The stepwise process of oocyte IVM is actively being explored with many promising studies working to achieve this critical milestone [39]. In vitro maturation is not yet validated for naturally existing human oocytes and further investigation is encouraged to develop clinically accepted IVM which will help to guide this important step in IVG and allow for iPSC-derived oogonia maturation as well.

These findings demonstrate that IVG research will likely yield a much better understanding of the process of natural gametogenesis and embryogenesis, possibly improving diagnostic and interventional tools for patients with infertility. For example, the successful differentiation of hiPSC to oocytes would provide innumerable cells, allowing for robust investigation within oocyte biology important to ART. This would accelerate studies on oocyte quality, including those on factors leading to ‘ageing’ (i.e., more error-prone meiosis and subsequent aneuploidy) or assessing the impact of cancer-curing medications upon oocyte function.

Oncofertility is an area of ART for which there would be great clinical benefit from IVG, allowing for rapid timely fertility preservation. This advance is especially pertinent for patients who require urgent gonadotoxic chemotherapy treatments or radiation therapy. Current oncofertility techniques for female patients, namely, oocyte and ovarian tissue cryopreservation, have limitations due to the invasive, expensive, and labor-intensive processes of obtaining the cells or tissue. Oocyte cryopreservation requires up to two weeks of ovarian stimulation medications including a low embedded risk of ovarian hyperstimulation which could further delay onset of chemotherapy followed by an oocyte retrieval procedure. Ovarian cryopreservation requires laparoscopic resection of ovarian tissue. Both require time and stable medical health as well as avoidance of ovarian hyperstimulation for a procedure or surgery to take place. Additionally, oocyte cryopreservation can only be performed in post-pubertal females and tissue cryopreservation would require either IVM technology, which currently does not fully exist or reimplantation of the ovarian tissue back into the patient (orthotopic transplantation) when the patient is ready for childbearing. There is a relevant concern that this has the potential to reimplant malignant cells back into the body. Additionally, IVG may allow the possibility to restore fertility following gonadotoxic chemoradiation therapy. The effect of such oncologic treatments on the ability of dermal fibroblasts to be used to form iPSC has not yet been studied but offers potential advantages which no other current oncofertility fertility preservation method currently provides.

IVG research also has the potential to contribute to modeling human embryo development and implantation, which is difficult to study given current restrictions and regulations on embryo research. One example of the way IVG is already advancing this research is through its use in the development of a microfluidic device that is expanding our understanding of embryo implantation and has the potential to aid in conditions like recurrent pregnancy loss [40]. The microfluidic device has the benefit of allowing the development of embryonic-like sac structures that are not capable of developing into a human fetus and therefore may be exempt from the 14-day research restriction. The device is highly scalable and consistent, allowing for higher throughput and precise research, including that of the effect of drugs or toxins on embryogenesis [40].

One unique future application of IVG is the potential to genetically modify hiPSCs from patients with medical conditions caused by specific genetic mutations that they wish not to pass on to their offspring. This could be especially relevant to cancer survivor patients whose malignancies have a specific genetic underpinning. Even without gene-editing technology such as CRISPR, there would be the availability to create a much larger pool of embryos than current IVF practice. From this much larger pool, preimplantation genetic testing for monogenic conditions (PGT-M) could be performed. This allows for embryo selection without the risk of having a child who has the condition in question [41]. The same benefit could help to solve one of IVF’s greatest challenges: that of helping women beyond their reproductive years create a healthy, genetically related embryo.

Lastly, and perhaps the most groundbreaking aspect of IVG’s potential, is its ability to shift the paradigm of reproduction at its core—that one ‘male’ and one ‘female’ are required to create new life. That is, for example, if both oogonia and spermatogonia are achievable via fibroblasts from skin punch biopsies of either sex, this could allow for same- sex couples to have a child that is genetically related to both parents. Currently, only a mouse model has demonstrated successful live birth in using two male gametes (sperm and oocyte) both originally from male mice ESCs, although spermatogonia have not been derived from a female mouse, but this work is ongoing [42,43,44,45]. Furthermore, an embryo could also hypothetically be made from a single biologic parent if both oocyte and spermatocyte were formed from the same person, as well as more than two biologic parents if two or more cycles of IVG were completed. Beyond the current biological and regulatory roadblocks to IVG, the future clinical applications of IVG and its derived research hold immeasurable potential while continuing to pose ethical concerns of the human application of IVG [46].

## 6. Ethical Considerations before Implementing IVG

At this point, IVG is lacking biologic safety data. There is currently no evidence that oogonia created from hiPSC are biologically equivalent and can produce healthy human fetuses. In fact, the field is so new that we do not yet have clarity on what specific, presumably genetic, testing might provide regarding sufficient evidence of their safety and if they would not create a fetus at risk of being born with significant comorbidities. This uncertainty regarding potential consequences of IVG is certainly a risk and preliminary animal model studies are actively being explored. Following animal study validation studies, it is likely that, similar to the first women who agreed to trial IVF pregnancies in the late 1970s and early 1980s, patients who may benefit the most from IVG would be agreeable to assuming the potential risk [47]. The ability to complete oocyte developmental competence via IVM in rOvarioids entirely ex vivo as opposed to requiring transplantation back into a live mouse gonad shows potential for mitigating future IVG risks [48]. The present-day ‘early’ expectations from IVG are likely to undergo a similar evolution in the definition of success that initially occurred in IVF. It has taken 40 years to appreciate what defines a ‘success’ in IVF. The aspirational outcome of IVF has shifted from its initial goal of clinical pregnancy in the early days, to live birth, to producing a single healthy newborn. Tomorrow’s definition of IVF may be a healthy baby from less than healthy gametes or from somatic cells via IVG.

Many people may utilize IVG to allow for a child in scenarios where there is no other way to obtain a genetically related gamete including: (1) within a same-sex couple, (2) patients beyond their reproductive years who do not desire oocyte donation, or (3) primary ovarian insufficiency/azoospermia in cancer survivors or due to other medical conditions or injury. In these scenarios, they have no alternative of having a biologically related child. One may compare these pregnancies in terms of the potential risk of issues to the child to that in a very young, or medically and/or mentally unwell mother. In these circumstances, the alternative is for the child to have never been born [47].

Concerns have been raised regarding the potential for IVG to promote the push for ‘designer babies.’ It is quite possible IVG could support this science, but this approach is already commercially available in some form through the sale of donor oocyte and donor sperm. Currently, infertile couples may purchase donor oocytes and sperm and do so while selecting certain traits of the donor including physical, ethnic, and even those relating to personality and intelligence.

One fear with IVG lies in how much less invasive a skin biopsy is than oocyte retrieval or sperm specimen collection, perhaps leading to a broader range of the population being willing to sell their own skin sample or to have it taken without consent. Since there is technology to duplicate stem cells without differentiating them [49], there would be the potential for an infinite number of offspring from one highly sought-after source, increasing the risk for consanguinity. Laws and regulations would need to be established to outline who had rights to use others’ stem cells if deemed genetically desirable. Interestingly, although other countries have legal limits on how many offspring can be the result of one donor, no such laws exist in the US. For the sake of reduction of risk of recessive gene- related diseases, the American Society of Reproductive Medicine (ASRM) suggests a maximum of 25 offspring per population of 800,000 [50].

There remains a risk that for an individual with desirable genes, skin biopsies could be theoretically ‘stolen’ from those who are not seeking such offspring, including at time of death. Although controversial, this is not an entirely new issue, as posthumous sperm retrieval already exists and has been used to father children [51,52].

As previously mentioned, IVG challenges one of the most core concepts of reproduction: that a fetus is created from one male-derived gamete and one female-derived gamete. With IVG, potentially a single person or groups of individuals could produce a child together (for example, with a mix of genes from four different gametes by using the hESCs from two embryos, with each of the two embryos made from two other in vitro generated stem cells). In this example, essentially the four individuals would be the grandparents of the one embryo, skipping a generation. If the technology was determined to be safe, then to disallow an embryo to be made in such a way would be to claim that only persons who abide by monogamy and not polygamy or single parenting by choice have rights to make a genetically related child. For a baby to intentionally have more than two biological parents is a progressive and controversial idea that requires the ethical consideration of the social and psychologic impact to those offspring. It is worth noting, however, that polygamy is widely accepted in many cultures, and children are commonly raised by people other than their biological parents, including grandparents, same-sex couples, and unrelated guardians.

In American culture, the normative standard of a family is a heterosexual couple with biologically related children. This highlights one of the inequities that exists for same-sex partners, contributing to the social discrimination of viewing these parents and their children as less than a family. Most states in the US have significant legal barriers to same-sex couples being the legal parents of their child. One can imagine that the formation of genetically related embryos may change the legal context in which these couples can exist, lessening the social stigma and potentially leading to greater social acceptance and support [53].

Regarding solo reproduction, this approach uniquely allows for genetic shuffling within the one person’s alleles through meiosis to form each gamete needed to produce the embryo. Therefore, the child would not be a clone of their parent but would be the equivalent of identical twins hypothetically conceiving a child together. Importantly, however, this approach would increase the risk of specific recessive genetic mutations resulting in disease in 25% of embryos. This is one of the several reasons that IVG may not fully replace gamete donation [54].

What makes the use of IVG for same-sex couples, partnerships of more than two people, and solo reproduction different is that its use is for social reasons as opposed to its use in oncofertility. The latter provides a treatment for a medical condition that caused infertility so severe that IVF would not be a viable option and is therefore understood as a ‘necessary’ therapeutic medical intervention. This is consistent with the standard goal of medicine: to improve patient health and cure disease. Currently, we allow all ART interventions to be used for medical and social reasons, including insemination with a deceased partner (posthumous reproduction). One could easily claim that in such circumstances, a person could choose to procreate with another live person, and as such, it is not truly medically necessary.

For those who argue against the social use of IVG claiming it is ‘unnatural’: would it be unnatural for a human to live in an artificially created environment in space? Yes, but for the interest of progress and the future of mankind, we have allowed this pursuit and have made significant strides in this field. Of course, for IVG research to continue to progress, policymakers must consider the ethical, legal, and social consequences to human reproduction and society at large. One such organization is the ISSCR, who, as previously mentioned, recently published updated guidelines that allow for more flexibility within stem cell research by expanding to approve the future study of IVG and its potential future use for infertility if safety and ethical issues are resolved (Table 2) [28].

## 7. Conclusions

Forty-five years have passed since Lesley Brown bravely gave birth to her daughter, Louise, the first baby conceived by IVF, and shocked the world by its novelty. Today, the apprehension around the ‘test-tube baby’ has passed and the percentage of babies born from IVF each year is rapidly increasing. The science of reproductive medicine continues to evolve and IVG is one of the most exciting areas that has largely developed over the past decade. The excitement within reproductive endocrinology and infertility regarding progression to the future clinical use of IVG is understood across the field.

IVG has the opportunity to address the limited reproductive potential of millions of cancer survivors in addition to the other unique patient populations it may serve. With approximately 1.9 million people diagnosed with cancer per year in the US and an estimated 26 million cancer survivors by 2040, IVG could have wide and lasting impact on cancer survivors [1,3]. Some prognosticate this technology to be introduced to patients within 10 to 20 years, depending upon the ethical, regulatory, and legal barriers which arise. Given the current promising mouse model results with the birth of pups from IVG from tail-tip fibroblasts and ongoing human studies utilizing skin biopsy fibroblasts, there is good reason for the enthusiasm this field is gaining. This research would not only benefit fertility preservation via IVF, but would surely improve our understanding of embryonic development, implantation, and genetic/epigenetic influences upon human development. There are many scientific barriers remaining prior to the application of IVG use for human reproduction. These areas of research include the maturation of human gametes from early stages to a stage in which fertilization can occur, strengthening the genetic and epigenetic integrity of the cells at each stage, testing embryo quality and later, the health of the live births of primate-derived embryos. While the ethical and legal barriers reviewed cannot be addressed in the laboratory, there are regulating bodies diligently working to address many of the ethical issues raised in this article. Many patients may benefit from the fruition of IVG technology, with cancer survivors of reproductive age being chief among them.

## Figures and Tables

**Table 1:** Stem Cell Terminology *.

**Stem Cells** (SC)—are unspecialized and retain the ability to differentiate into any cell of an organism along with the ability to regenerate
**Embryonic Stem Cells** (ESC)—stem cells obtained from the inner cell mass of an embryo in the blastocyst stage
**Pluripotent Stem Cells** (PSC)—form cells of all germ layers but not extraembryonic structures, such as the placenta (for e.g., embryonic stem cells)
**Multipotent Stem Cells** (MSC)—can specialize in discrete cells of specific cell lineages (for e.g., hematopoietic stem cells)
**Oligopotent Stem Cells** (OSC)—can differentiate into several but not all cell types. (for e.g., myeloid stem cell can divide into white blood cells but not red blood cells)
**Unipotent Stem Cells** (USC)—retain limited differentiation but unlimited division (for e.g., dermatocytes)
**Induced Pluripotent Stem Cells** (iPSC)—are somatic cells that are reprogrammed to regain the differentiation capacity of **an** embryonic stem cell, considered to be equivalent to embryonic stem cells, but can be patient-specific
**Primordial Germ Cells** (PGC)—**primary** undifferentiated stem cell type that will form a gamete, either oogonia or spermatogonia
**Induced Primordial Germ Cell-Like Cells** (iPGCLCs)—PGCs derived from iPSCs, equivalent to PGCs, but developed in vitro

* All terms may begin with an ‘h’ or ‘m’ indicating ‘human’ or ‘mouse’, respectively.

**Table 2:** Main Barriers to Human Application.

**Scientific:** In vitro formation of mature human oocytes and sperm (currently only imma-ture oogonia have been derived from fibroblasts) Creation of human artificial ovary and testes environments via formation of supportive cells from iPSC (currently only mouse-derived somatic cell environments are available) Embryogenesis from fibroblast derived oogonia-like cells (embryos have cur-rently only been made from mouse-derived cells that have resulted in live pups) Biologic integrity assessment of each stage of maturation, will likely be through genetic and epigenetic markers (such markers are yet to be determined) Safety testing for patients with cancer (including whether biopsy is taken before or after cancer treatment) **Ethical:** Consensus needs to be determined as to the limits of IVG in terms of ethical principal of: ⚬ Extension of in vitro embryo development beyond 14 days ⚬ General Use in any circumstance to form a human life ⚬ Use for biologically related offspring for homosexual couples ⚬ Use for solo-parenting ⚬ Use of multi-plex parenting ⚬ Selection of offspring without genetic conditions ⚬ Regulation of the biopsy source ▪ Number of offspring from one individual ▪ Consent, including posthumous biopsy

## Data Availability

Not applicable.
